# Novel high-quality and reality biomaterial as a kidney surgery simulation model

**DOI:** 10.1371/journal.pone.0263179

**Published:** 2022-02-17

**Authors:** Taro Kubo, Tatsuya Takayama, Akira Fujisaki, Shigeru Nakamura, Takumi Teratani, Naohiro Sata, Joji Kitayama, Hideo Nakai, Daiki Iwami, Tetsuya Fujimura

**Affiliations:** 1 Department of Urology, Jichi Medical University, Tochigi, Japan; 2 Division of Renal Surgery and Transplantation, Jichi Medical University, Tochigi, Japan; 3 Department of Pediatric Urology, Children’s Medical Centre Tochigi, Jichi Medical University, Tochigi, Japan; 4 Department of Surgery, Division of Gastroenterological, General and Transplant Surgery, Jichi Medical University, Tochigi, Japan; 5 Division of Translational Research, Jichi Medical University, Tochigi, Japan; University of Cambridge, UNITED KINGDOM

## Abstract

Surgical training using live animals such as pigs is one of the best ways of achieving skilled techniques and fostering confidence in preclinical medical students and surgeon trainees. However, due to animal welfare ethics, laboratory animals’ usage for training should be kept to a minimum. We have developed a novel kidney organ model utilizing a simple procedure in which the kidney is first refluxed with N-vinyl-2-pyrrolidone (NVP) solution for 1 hour in its bath, followed by permeation for 23 hours, with a subsequent freshwater refluxed for 48 hours in the washing step. Surgical simulation of the prepared kidney model (NVP-fixed kidney) was compared with three types of other basic known simulation models (fresh kidney, freeze-thaw kidney, and FA-fixed kidney) by various evaluations. We found the NVP-fixed kidney to mimicked fresh kidney function the most, pertaining to the hardness, and strength of the renal parenchyma. Moreover, the NVP-fixed kidney demonstrated successful blood-like fluids perfusion and electrocautery. Further, we confirmed that surgical training could be performed under conditions closer to actual clinical practice. Our findings suggest that our model does not only contribute to improving surgical skills but also inspires the utilization of otherwise, discarded inedible livestock organs as models for surgical training.

## Introduction

Lack of basic physician knowledge and skills often causes malpractice. Therefore, the physician must acquire basic knowledge and further improve skills. Medical care has undergone a major paradigm shift since malpractice has been reported to cause serious health damage to patients, and there is a strong global demand for safety and quality assurance [1, 2]. For medical staff to master clinical procedures, acquire the desired operate with confidence, education, and training are critical ventures [2], which could be categorized into two. One is “Knowledge,” and the other is “Technical Skill”. The former is for learning scholarship through lectures such as anatomy, surgical procedures, and the characteristics of the equipment; the latter is for acquiring skills through a practical experience such as surgical procedures and equipment usage [3–5]. However, advances in medical technology have made technical skills more specialized and limited.

The acquisition of technical skills for the surgical treatment of renal cell carcinoma (RCC) is one of the more specialized and limited fields. In comparison with “radical nephrectomy (RN)” with “partial nephrectomy (PN)” in the treatment of small (<4 cm) RCC renal cancer, there is no difference in the monitoring and management [6–8]. RN reduces overall renal function, whereas PN improves the maintenance of renal function [9, 10]. Besides, performing PN to preserve renal function significantly reduces the patients’ overall mortality and cardiovascular complications, compared to RN [11–13]. Therefore, PN is considered the gold standard in the treatment of small renal tumors. During the PN surgical procedure, first, the blood flow to the affected kidney is temporarily blocked. Next, the tumor containing a surrounding normal tissue is excised, the cross-section of the extracted kidney is sutured for temporary hemostasis, and finally, blood flow is resumed. This procedure is a highly advanced technique that requires the simultaneous operation of “total cancer removal” and “as little removal of the normal kidney as possible” with the minimum kidney ischemic time [14]. We reported earlier that the 3-dimensional (3D) model of the renal tumor was useful for pre- and intra-operative simulations in robot-assisted PN [15], but training to improve the technical skill for the operations involved is still insufficient.

So far, when training in surgical procedures, artificial models, resembling organs and tissues, are generated from flexible materials such as silicone or large laboratory animals such as pigs that are anatomically similar to humans are used. Although artificial models made of silicon are industrially mass-produced and care available in abundance for utilization in surgical training, technically, it is difficult and expensive to use it to create an organ with such a complex structure and function as the kidney. Training with laboratory animals allows the individual to practice and gain experience that most closely resembles the clinical setting. Although it is necessary to set up a facility to carry out such experiments, a large amount of cost is required to meet the demand. Furthermore, from the viewpoint of animal welfare, it is desirable to minimize the number of training with laboratory animals. As a result, there has been a shift to training using the cadaver. In Cadaver Surgical Training, the student has the advantage of being able to manipulate the organ(s) directly using actual surgical instruments. Still, there are challenges in securing enough specimens and establishing organ fixation methods. Therefore, in order to acquire sufficient surgical skills in medical education and clinical practice, it is necessary to create a training model using completely new materials.

In recent years, cadaver’s fixation method using NVP has received increased attention from the research community [16]. N-vinyl-2-pyrrolidone (NVP) is a monomeric form of hydrophilic polyvinylpyrrolidone [17] and a highly safe compound used in the manufacture of contact lenses, artificial teeth, absorbable surgical threads, disinfectants, and cosmetics. From the perspective of animal welfare, it is necessary to make effective use of inedible organs of livestock. Therefore, we focused on N-vinyl-2-pyrrolidone (NVP) and livestock organs. Most of the organs that are not used for food of livestock animals are discarded, and effective use is required. This paper presents the novel method of constructing a training model that approximates a real organ and its usefulness by an entirely new organ fixation method using NVP.

## Materials and methods

### Isolated kidney

An intact whole kidney sample of Göttingen Minipig (6–9 kg in weight) with the renal artery, vein, and ureter preserved as much as possible, sold by Oriental Yeast Co., Tokyo, Japan, was purchased and used for the experiment. Additionally, the extension tube (Nipro Co., Tokyo, Japan) was cannulated into the lumen of the renal artery and vein of the kidney. After saline (0.9 percent sodium chloride; Otsuka Pharmaceutical Co., Tokushima, Japan) was manually injected from the tube to remove blood components in the tissue, UW solution (University of Wisconsin cold preservation/storage solution; Astellas Co., Tokyo, Japan) was injected for kidney preservation at cold storage state. At the start of the simulation experiments, the saline was injected into the renal artery and ligated the ureter with silk thread after washing of the UW solution. We used a total of 26 Göttingen Minipig kidneys, 4 for altitude measurements, 20 for strength measurement, and 2 for the reflux tests.

### Preparation of kidney for the surgical training

**Fig 1:**
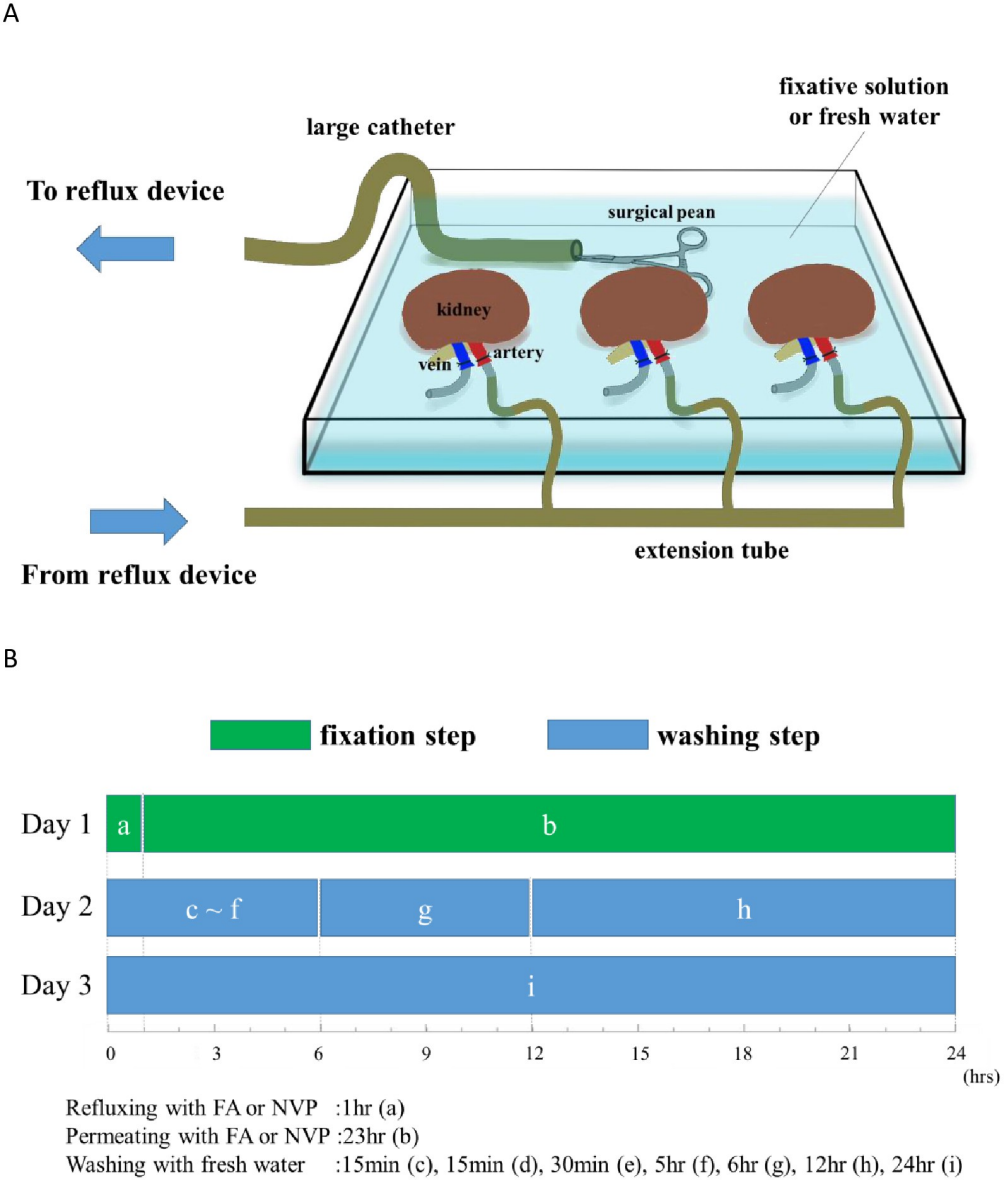
Circulating system and schedule for preparing the surgical training kidney. (A) Circulating system: The extension tube was cannulated into the lumen of the renal artery and vein of the pig kidney; the kidney was submerged the fixative solution bath. The reflux system was constructed to recirculate in its bath. The fixative solution was continuously collected from the tip of a large catheter, which was secured with a surgical pean (hemostat) that typically controls bleeding. A large catheter was connected to the extension tube, and the fixative solution was continuously refluxed into the renal artery. The fixative solution that had cycled through the kidneys was drained continuously from the renal vein to its bath. The drained fixative solution was collected from the large catheter again. (B) Schedule: The green bar is the fixation step. The blue bar is the washing step. a) is refluxed time. b) is permeated time. From c) to i) are replaced time. Fixation was first refluxed with FA or NVP fixative for 1 hour in its bath, then, by permeation for 23 hours. Following continuous freshwater reflux in the washing step, it was replaced periodically at 15 minutes, 30 minutes, 60 minutes, 6 hours, 12 hours, and finally 24 hours.

Four types of kidney samples were prepared, as follows: fresh kidney, freeze-thaw kidney, formaldehyde (FA)-fixed kidney, and N-vinyl-2-pyrrolidone (NVP)-fixed kidney. For FA-fixed kidney, 10% neutral buffered formalin solution (Wako Pure Industries, Tokyo, Japan), which is widely used for fixing pathological tissues, was used. The NVP-fixed kidney was prepared by mixing NVP (Preserve^®^, Nippon Medical & Chemical Instruments Co., Osaka, Japan) and 100% ethanol (Wako Pure Industries, Tokyo, Japan) at a ratio of 50% by weight (w/v%), and the solution 5-times with saline. The freeze-thaw kidney, which was stored at −80°C was thawed in the refrigerator at 4°C to avoid damage to the renal capsule and tissue. As FA- and NVP-fixed kidneys reflux device, MASTER flex L/S^®^ EW-07559-10 (Cole-Parmer Instrument Co., Illinois, USA) was used. Kidney fixation was first refluxed with FA or NVP fixative for 1 hour in its bath, followed by permeation for 23 hours, and then the kidneys were refluxed with freshwater for 48 hours as the washing step. The injection rate of fixative and water was 50 ml/min, and the reflux system was constructed to recirculate in its bath (Fig 1A). The freshwater was refluxed continuously in the washing step, but it was replaced with new water at time intervals of 15 minutes, 30 minutes, 1 hour, 6 hours, 12 hours, and finally, 24 hours in the washing step (Fig 1B).

### Evaluation of surgical training kidney

In the clinical experimental practice, PN was performed according to the procedure shown in S1 Fig. First, the visual and pathological findings of kidney samples were evaluated. Next, the hardness and strength of freeze-thaw kidney, FA-fixed kidney, and NVP-fixed kidney, were evaluated to assess if the kidney samples have condition approximated to that of a fresh kidney.

#### Hardness evaluation

**Fig 2:**
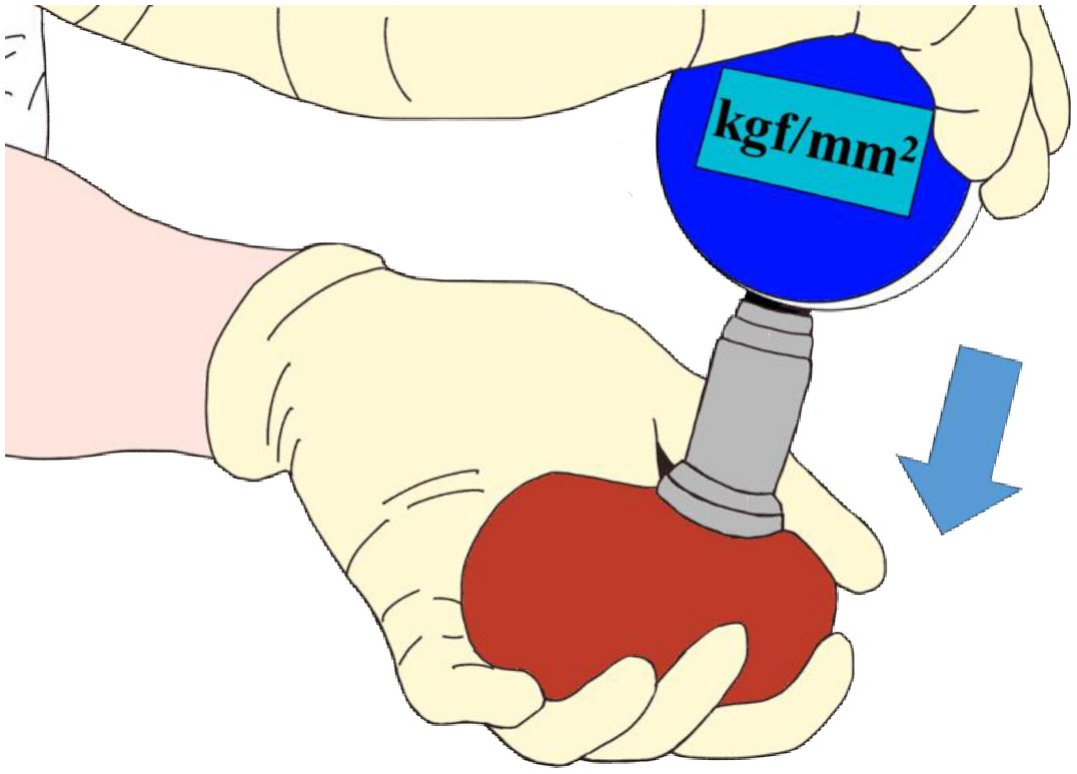
Hardness evaluation. A view of a hardness meter being pressed against the center of the kidney.

Using a hardness meter (NEUTONE TDM-ZK1 (RB), TRY-ALL Co., Chiba, Japan), the hardness of each renal carrier was evaluated (Fig 2). The hardness of kidney samples was measured at 1 hour, 2 hours, 3 hours, 6 hours, 24 hours, 48 hours, and 72 hours of washing steps. Each time, the hardness was measured three times at the same location in the central part of all kidney samples, and the average value was calculated.

#### Strength evaluation

**Fig 3:**
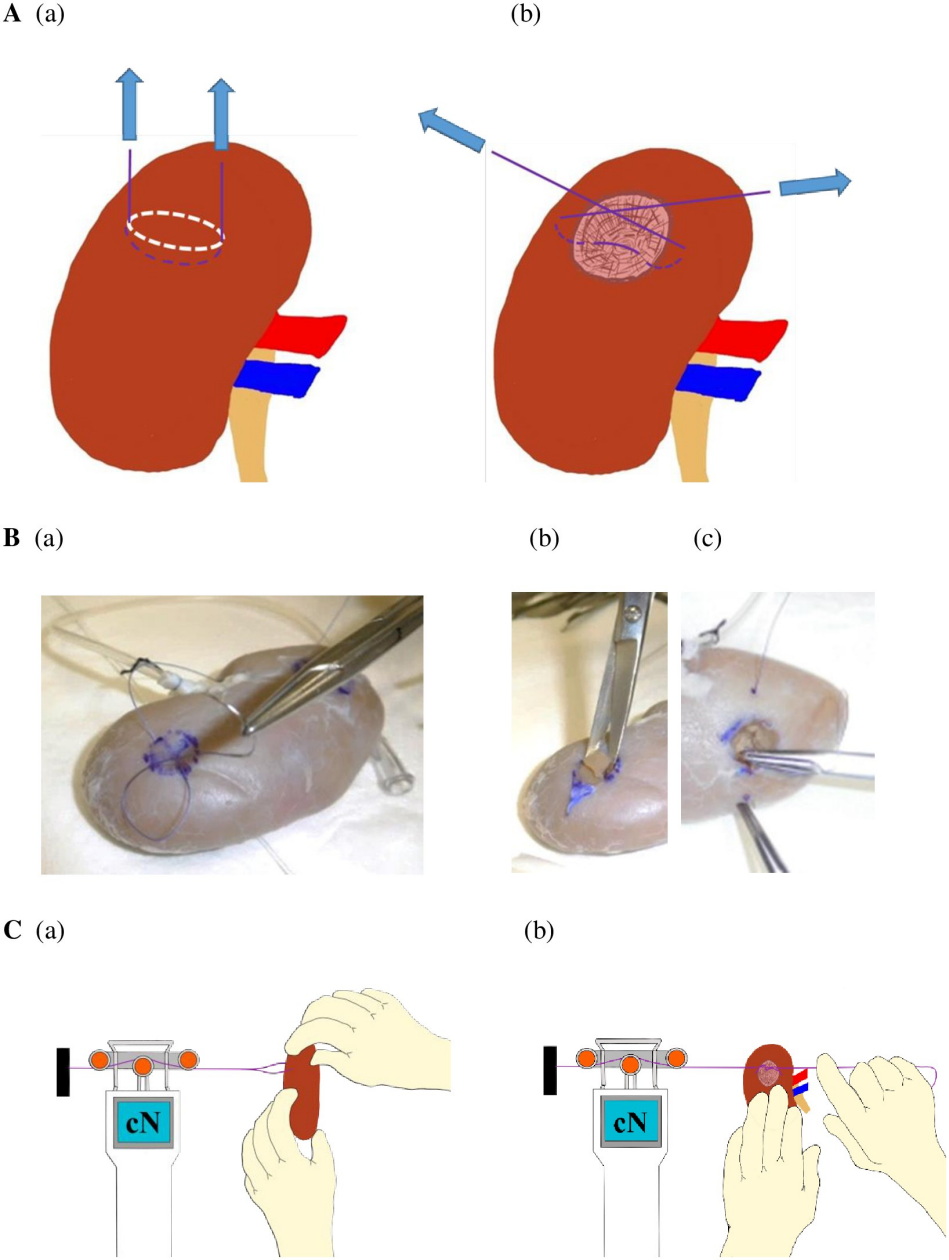
Strength evaluation. (A) Schematics to evaluate the strength of each kidney sample during towing (a) and during ligation (b). (B) The procedure of suturing. The drawing of a circle and the moving of the needle according to its diameter (a) resecting the renal parenchyma in a hemispherical shape (b) and moving the needle to close the defect of the renal parenchyma (c). (C) The procedure of towing (a) and ligation (b). See S1 Movie.

The tensiometer (TX-1000-1, Tensitron, Colorado, USA) was used to evaluate the strength of each kidney samples during towing [Fig 3A(a)] and during ligation [Fig 3A(b)]. The tension was measured five times for each kidney samples. As the suture thread, 3–0 vicryl CR SH-1 (Ethicon, Ohio, USA) with a 22 mm needle, which is commonly used in the medical field, was used.

For measuring towing capacity, at first, we placed markings (dots) with a width of 10 mm on the top of each kidney samples and a width of 15 mm on the bottom and moved the needle according to their distance intervals [Fig 3B(a)]. Next, sutures were performed at both ends of the sutured tissue, and were attached to a tensiometer. Finally, the tensiometer was fixed on the table; only the kidney sample was pulled, then tension was applied to the sutures. Next, traction was continued until the renal parenchyma was torn. The stress when the stitch tears the tissue was measured, as the stress retention capacity [Fig 3C(a) and S1 Movie].

For measuring ligation strength, a circle having a diameter of 10–15 mm was drawn on the upper part of each kidney samples, and a hemispherical part corresponding to the circle was resected from the renal parenchyma to prepare a model [Fig 3B(b)]. Next, a mark was placed where the width of the ligature was twice the diameter of the circle, and the needle was carried from the mark through the center of the defect to the mark on the opposite side [Fig 3B(c)]. Finally, the sutures were crossed, one end of the suture was fixed to the tensiometer, and then the experimenter pulled the suture [Fig 3C(b) and S2 Movie].

### Evaluation of surgery using blood-like fluids return and electrocautery

Changes in coloration of renal parenchyma were evaluated by spontaneous drops of saline (blood-like fluids) stained in the renal arteries of NVP-fixed kidneys in a 70 cm^3^ water column. The kidneys were processed using the electrocautery system according to PN and evaluated for differences from the surgery of living pigs.

### Statistical analysis

Data are represented as means ± SEM. Results were analyzed using a two-tailed Student’s t-test to assess statistically significant correlations between two measured variables. A *P* value of <0.05 was considered significant.

## Results

### Visual and pathological findings of surgical training kidney

**Fig 4:**
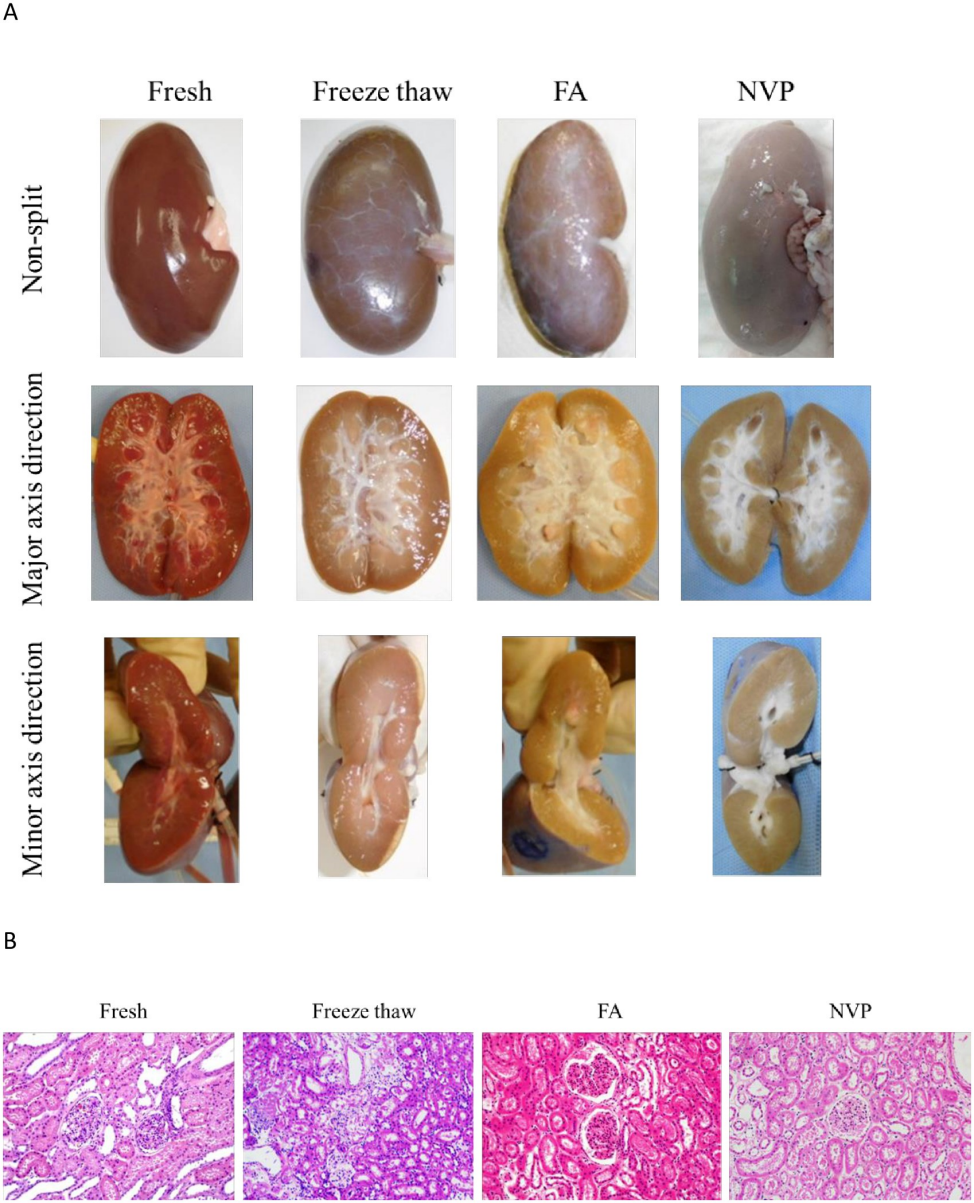
Visual and pathological findings of surgical training kidney. (A) Macroscopic findings of fresh kidney, freeze-thaw kidney, FA-fixed kidney and NVP-fixed kidney. The upper section is a finding without an incision. The middle section is major axis and the lower section is minor axis direction. (B) Light microscopic findings of fresh kidney, freeze-thaw kidney, FA-fixed kidney, and NVP-fixed kidney, which were hematoxylin and eosin stained.

At first, the color tone of each kidney samples was evaluated. The fresh kidney and freeze-thaw kidney maintained color tones similar to that of the kidney received immediately after removal from the organism. On the other hand, FA- and NVP-fixed kidneys changed to a cloudy white color (Fig 4A, top). For this reason, the blood component in the FA- and NVP-fixed kidneys were washed thoroughly by the circulation of the fix-solution. Subsequently, our macroscopic findings based on the evaluation of the split surface in the major axis direction and the minor axis direction revealed destruction of the structures of the renal cortex and renal medulla of the freeze-thaw kidney but were preserved in fresh-, FA- and NVP-fixed kidneys (Fig 4A, middle and bottom). Finally, the pathological findings of each kidney evaluation demonstrated the occurrence of structural disruption in the freeze-thaw kidney, whereas the cell arrangement was highly maintained in the fresh- and FA-fixed kidneys; meanwhile, a very slight structural disruption was observed in the vascular endothelial cells for NVP-fixed kidney sample (Fig 4B).

### Evaluation of hardness for kidney samples

**Fig 5:**
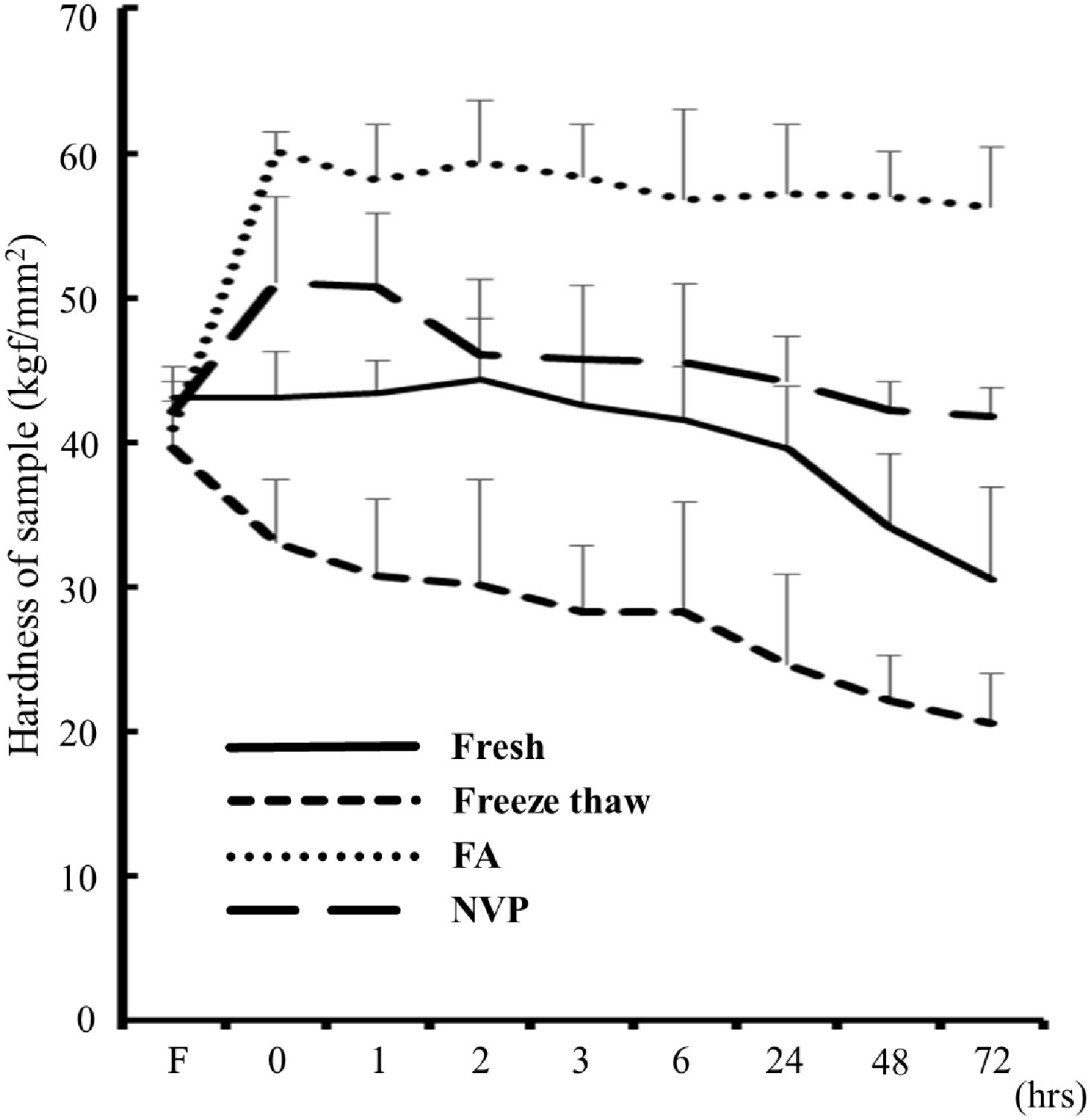
Evaluation of hardness for kidney samples. The graph shows the hardness of the renal parenchyma, fresh carriers (solid line), freeze-thaw carriers (dashed line), FA-fixed carriers (dotted line), and NVP-fixed carriers (long dashed line). Compared with fresh kidney samples, the hardness of the NVP-fixed kidney sample, after 72 hours of washed, revealed no significant difference.

The hardness of each of the three kidneys treated groups were as follows: (1) FA-fixed kidney, (2) fresh- and NVP-fixed kidneys, and (3) freeze-thaw kidney (Fig 5). The hardness of the fresh kidney was maintained up to 6 hours by injecting water into the artery, but decreased after 24 hours, declined to ~70.8% at 72 hours. The FA-fixed kidney, however, increased to ~146.6% immediately after the end of fixation and remained almost unaltered after intra-arterial water injection. On the other hand, the hardness of the NVP-fixed kidney increased to about 121.4% immediately after fixation, which almost paralleled the behavior trend of the FA-fixed kidney. However, by 48 hours after injecting water into the kidney arteries, it decreased in hardness in the same manner as the fresh kidney and was maintained up to 72 hours. Thus, these results showed that, compared with fresh kidney, the hardness of the NVP-fixed kidney, after 72 hours washed, showed no significant difference than the fresh kidney, while the dramatic difference was observable with the freeze-thaw- and FA-fixed kidneys. Therefore, the hardness of the NVP-fixed kidney was considered to be the best wash time of 48–72 hours period suitable as the surgical training kidney model.

### Measuring of kidney samples strength

#### Towing test outcomes

When the marking was 10 mm, the tension during traction was 383.25 ± 144.89 cN, 139.75 ± 49.26 cN, 149.25 ± 55.76 cN, and 347 ± 38.45 cN for the fresh, freeze-thaw, FA-fixed, and NVP-fixed kidney, respectively. When the marking was 15 mm, the tension during traction was 589 ± 162.94 cN, 253.75 ± 57.2 cN, 22 ± 7.3 cN, and 408.75 ± 55.77 cN for the fresh, freeze-thaw, FA-fixed, and NVP-fixed kidney, respectively. In this result, an NVP-fixed kidney was found to have the most suitable towing value close to the fresh kidney.

#### Suturing test outcomes

In the case of the hemisphere-deficient renal parenchyma diameter was 10 mm, the tension at ligation was 276.25 ± 55.13 cN, 108.75 ± 51.27 cN, 34.25 ± 14.01 cN, and 292.75 ± 47.08 cN for fresh, freeze-thaw, FA-fixed, and NVP-fixed parenchyma, respectively. For the 15 mm diameter, the tension at ligation was 210.75 ± 151.6 cN, 95.25 ± 8.58 cN, 21.5 ± 13.28 cN, and 147.75 ± 31.16 cN for fresh, freeze-thaw, FA-fixed, and NVP-fixed parenchyma, respectively. In particular, it was confirmed that the tissue of the FA-fixed kidney was disintegrated rather than torn when the suture was pulled. In these results, the NVP-fixed kidney was the closest to the fresh kidney in handling tension during both traction and suturing.

### The evaluation result of surgery using pseudo-blood return and electrocautery

**Fig 6:**
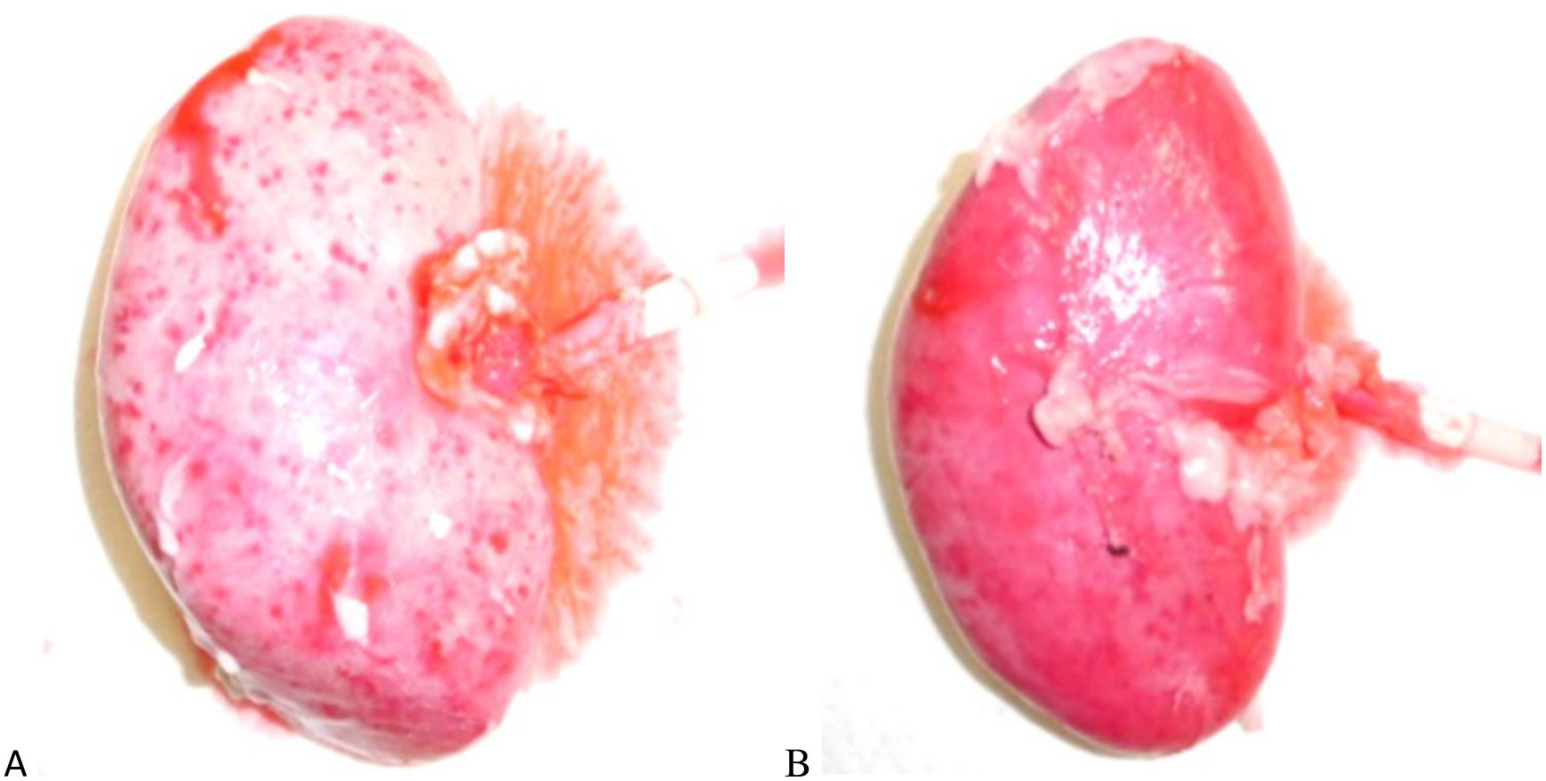
Evaluation of surgery to use blood-like fluids return. (A) Immediately after the infusion of blood-like fluids, the kidney became discolored in diffuse spots. (B) In about 5 minutes, the kidneys were uniformly discolored.

Finally, the effectiveness of the NVP-fixed kidney was evaluated for plausible use as a human clinical kidney device and in surgical operation protocols. When blood-like fluids flowed into the renal artery of the NVP-fixed kidney, the renal parenchyma turned red evenly over time, confirming the sustenance of the vasculature’s structure in the NVP-fixed kidney and the transport of liquid components in the vascular lumen (Fig 6 and S4 Movie). After resection of the renal parenchyma and suturing of the defect in this condition, the NVP-fixed kidney confirmed the sustained flow of blood-like fluids from the dissected section of the renal parenchyma. At an important point, the results confirmed the feasibility of a highly realistic surgical operation that closely resembles a PN in a living pig (S5 Movie). The incision of the NVP-fixed kidney, using electrocautery was similar to that of living pigs in terms of resistance at the time of incision and thermal degeneration of the cross-section (S6 Movie). In contrast, our data confirmed that the FA-fixed kidney performance was poor both in blood-like fluids flow and electrocautery incision. Hence, these findings confirmed that the NVP-fixed kidney was useful as training model for the incision and dissection of renal tissues using electrocautery because the vascular system was relatively well preserved.

## Discussion

To maintain or improve the skills of surgeons and train medical students, it is necessary to engage students in skilled practice to enable them to ultimately, perform as close to the actual clinical scenarios as possible. One of the many ways to accomplish these tasks is to create a training model that closely resembles an in vivo organ status. We have succeeded in producing a kidney training model that is very close to the fresh kidney. Our manufacturing method was quite simple. The kidneys were treated with NVP solution and simply perfused with water for an optimal time (48 hours) to wash out the NVP fixation and adjust the hardness of the kidneys as close to the fresh as possible.

Surgical training using the NVP-fixed kidney has several advantages. First, the hardness and strength of the NVP-fixed kidney are equivalent to those of the fresh kidney. Although there are several reports on the efficacy of a 10 mm long renal tumor model in surgical training with ablation therapy [18, 19], there has been no report that the hardness or strength of the training model has a significant influence on the accuracy of the procedure such as resection and ligation of renal parenchyma in PN. The freeze-thaw kidney showed a marked decrease in its hardness immediately after thawing. The hardness of the FA-fixed kidney increased markedly immediately after fixation, which remained in the hardened state even when water was refluxed. On the contrary, the hardness of the NVP-fixed kidney was adjusted to that of the living organ by constant water reflux (Fig 5). When the hardness decreased, as in the case of the freeze-thaw kidney, the ligature could be completed with less tension when performing the ligation. When the hardness increased, as in the case of the FA-fixed kidney, the tissue was torn by a small amount of tension when performing ligation, so that the ligature could not be completed (S3 Movie). Therefore, we envisaged that it would be challenging to perform highly accurate training employing both models. In recent years, cadaver’s fixation method using NVP has received increased attention from the research community [16]. To the best of our knowledge, this study is the first to demonstrate that NVP has medically useful properties. The first is “fixation” for the long-term preservation of organs, and the second is “hydration” for softening organs. Most famous cadaver’s fixation method using FA is Thiel’s method [20–22]. In that method, the ratio of FA to fixed tissue is approximately one-half to one-third, which is insufficient for the long-term preservation of organs. This study confirms that FA does not enhance hydration because it is a non-hydrophilic organic compound.

Secondly, the NVP-fixed kidney could be utilized for surgical training purposes due to its ability to mimic the condition of blood-like fluids reflux. Once the blood-like fluids flowed, it returned uniformly to the NVP-fixed kidney, as in the case of renal reperfusion during renal transplantation (Fig 6). The NVP-fixed kidney combined the optimal hardness of vessels with the anatomical vascular system, as shown by pathological findings (Fig 4B). Therefore, perfusion of simulated blood indeed allowed the training of highly realistic surgical procedures and is comparable to training using fresh kidneys (S4 Movie). We presumed that using NVP-fixed kidneys would provide a skilled practice for PN, as this organ model could be employed to deliver both ischemic and non-ischemic training. Non-ischemic training requires maintaining simulated blood perfusion (S5 Movie), whereas ischemic training requires an additional vascular clamping process, both demonstrated using this novel model.

Third, an electric scalpel is energized and works appropriately in the NVP-fixed kidney. The NVP- and FA-fixed kidneys are treated with fixative as well, but unlike the FA-fixed kidney, the electric scalpel worked properly and allowed for coagulation and dissection. This is the most critical and attractive feature of the NVP-fixed kidney (S6 Movie). Therefore it is possible to maximize the training effect by utilizing the NVP-fixed kidney regarding the technical approach for PN using a simulation model such as silicon, shown to exhibit effective performance [23–25].

Essential Skills in the Management of Surgical Cases (ESMC) recommends using living pigs to understand surgical approaches and improve basic surgical skills [26]. Training with living pigs is one of the best instructions currently conceivable, as they represent models which reproduce, in particular, renal anatomy, as well as physiology, as close enough to humans as possible. Our facility has been studying training methods for surgery with pigs for many years [27]. Box trainers, such as pulsation organ perfusion (POP) trainers (Optimist, Innsbruck, Austria), are used for training that could provide tactile feedback using real tissue and real equipment. Also, the preparation time is short, and both animal and good artificial materials could be used to compare outcomes. Training using POP trainers is an economical and efficient surgical training that can significantly reduce the number of animal experiments [28–30]. However, it is difficult to secure a large number of training animals because animals need to be killed only for limited surgical training purposes. By replacing the organs of livestock that are discarded because they are not typically used as edibles with training models, we believe that more laboratory animal lives could be saved. Furthermore, the NVP-fixed kidney could be stored for an extended period, and thus, a large number of training models could be secured.

Artificially manufactured tissue may be superior in terms of cost and ethics. However, kidney has a very complex structure and many current kidney models tend to ignore important microscopic details. Furthermore, our kidney model successfully preserved the vascular system, which can be used for on-pump surgery. If we can reuse the inedible organs of livestock designated for disposal, our manufacturing method will actually be less expensive than that of artificial kidneys. This technology is also expected to make effective use of inedible livestock organs that are usually discarded from the viewpoint of humane treatment of animals and their byproducts.

## Conclusions

**Fig 7:**
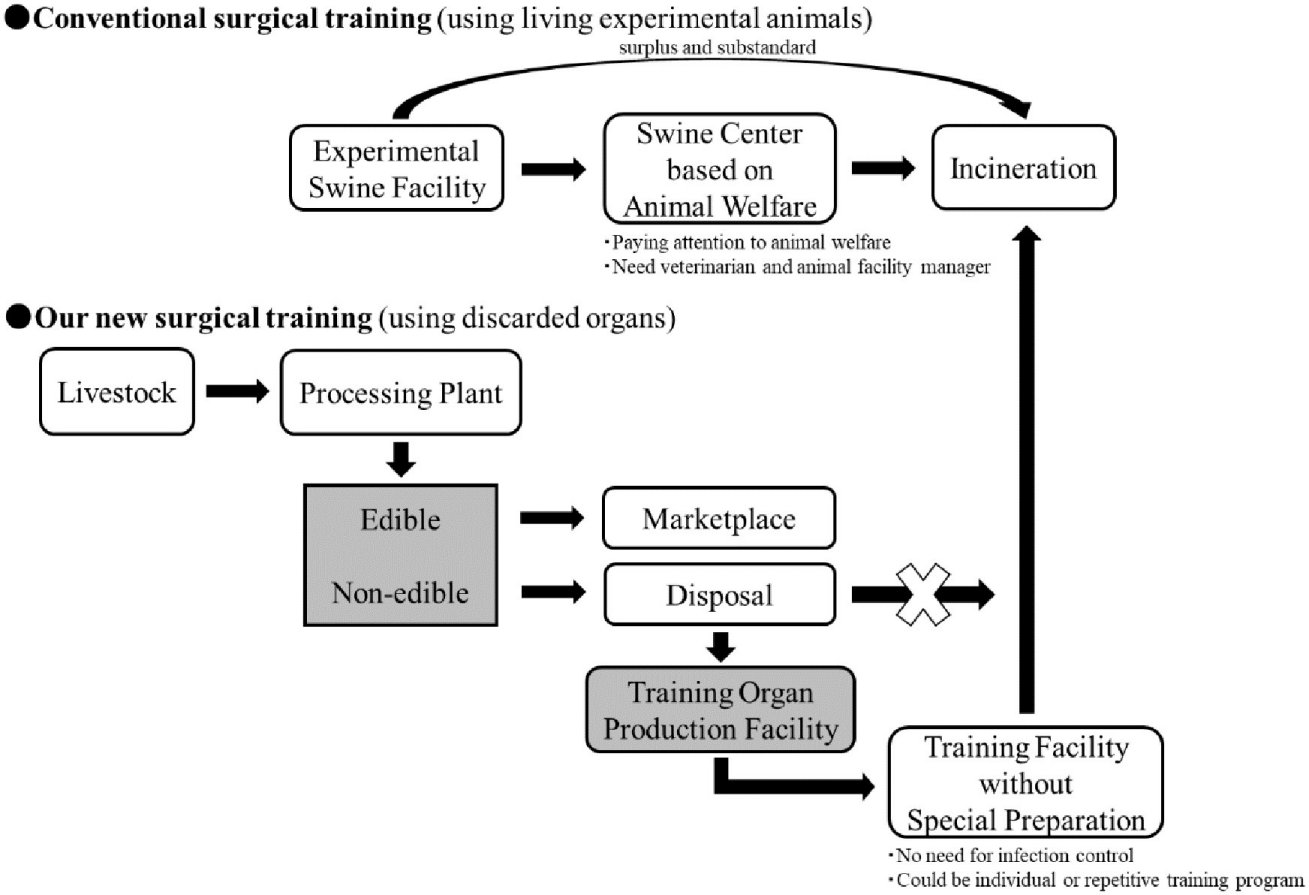
Concept of our new surgical training model. Standard advanced surgical training was performed using a living experimental animals, and multiple trainings are required to master the technique. Therefore, many living experimental animals was necessary. However, our new concept was recycled the waste organs at meat processing plants for surgical training materials, and it was possible to significantly reduce the number of the living experimental animals.

A new method for fixing an organ using a hydrophilic polymer has attracted attention recently [16, 31–33]. However, this is the first method to fix organs using hydrophilic polymeric compounds with a controlled reflux time with water. Our protocol is expected to achieve a significant innovation in cadaver fixation technology for embalming. Since it is a significant presumption that to secure arteriovenous for organ perfusion is critical, it is necessary to pay close attention not to damage the vascular system during organ harvesting. We have confirmed that the NVP fixation method is also significant in the liver (manuscript in preparation), and we are planning to use the same protocol for the fixation of other major organs in the future. In general, the handling of the undiluted NVP requires the same care as in FA [33]. Our protocol used diluted NVP, and since the organs are perfused with water even after NVP fixation, safety should be guaranteed. More importantly, conventional surgical training is performed using living experimental animals, but our new concept is recyclable for surgical training using a sample from discarded organs in meat processing plants (Fig 7). Thus, our model would undoubtedly contribute to a significant reduction in the utilization of numerous living animals with the associated cost-intensiveness.

In conclusion, our novel protocol suggested that for making surgical training models, strategic planning and exploiting novel materials could drive the medical technology industry to a position where organ models could be mass-produced. Yet it could mimic a real-life clinical scenario as much as possible to provide stability in the supply of training and a model as highly realistic as possible. This will contribute to the development of clinical skills and lead to further improvements in medical safety and quality.

## Supporting information

S1 Fig The surgical procedure of partial nephrectomy (PN). First, the blood flow to the affected kidney is temporarily blocked. Next, the tumor containing the surrounding normal tissue is excised, the cross-section of the extracted kidney is sutured hemostatically, and finally, blood flow is resumed. (a) Asterisk is renal tumors that protrude on the surface of the kidney. (b) The excised tumor containing the surrounding normal tissue. (c) Suturing of the cross-section of the extracted kidney. (d) Finished hemostasis of the defect.
(DOCX)

S1 Movie A video of towing. The suture was towed perpendicular to the renal capsule until the tissue (renal parenchyma) was torn (Strength evaluation of towing and ligation).
(MP4)

S2 Movie A video of ligation. The ligated suture was towed horizontally against the renal capsule until the tissue (renal parenchyma) was torn (Strength evaluation of towing and ligation).
(MP4)

S3 Movie A video of ligation; in the case of the FA-fixed kidney. The tissue (renal parenchyma) was torn by a small amount of tension when performing ligation (Strength evaluation of towing and ligation).
(MP4)

S4 Movie A video of blood-like fluids reflux. When blood-like fluids flowed into the renal artery, the renal parenchyma turned to the red evenly over time. Also, it continuously drained from the vein. (Evaluation of using the human clinical device and surgical operation protocol in the NVP-fixed kidney).
(MP4)

S5 Movie A video of PN. The resistance to incision and ligation closely resembles that of a living kidney. Persistent blood-like fluids infiltrate from the cross-section of the extracted kidney, confirming that it is not only tactile but also visually similar to the living organ. (Evaluation of using the human clinical device and surgical operation protocol in the NVP-fixed kidney).
(MP4)

S6 Movie A video demonstrating the use of the electrocautery equipment. The incision and thermal degeneration of the kidney using electrocautery was similar to that of the living organ. (Evaluation of using the human clinical device and surgical operation protocol in the NVP-fixed kidney).
(MP4)

## Abbreviations

FA: formaldehyde
NVP: N-vinyl-2-pyrrolidone
PN: partial nephrectomy
RCC: renal cell carcinoma
RN: radical nephrectomy
