# Supplementary figures and images for: Novel high-quality and reality biomaterial as a kidney surgery simulation model
Source: PLoS One. 2022 Feb 17;17(2):e0263179. doi: 10.1371/journal.pone.0263179 (PMC8853465; doi:10.1371/journal.pone.0263179)

**S1 Figure**

1. (b)


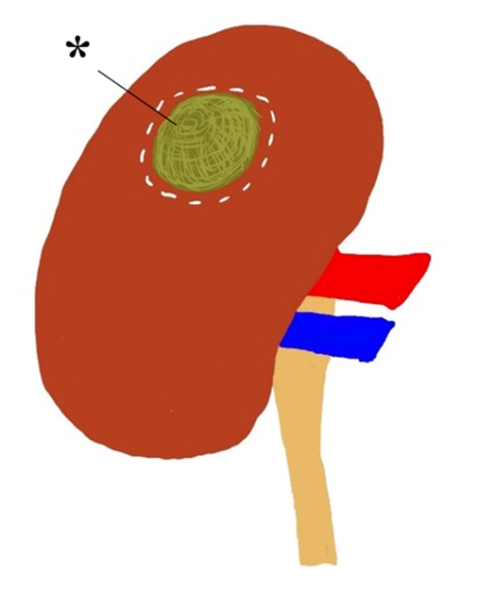
 　　
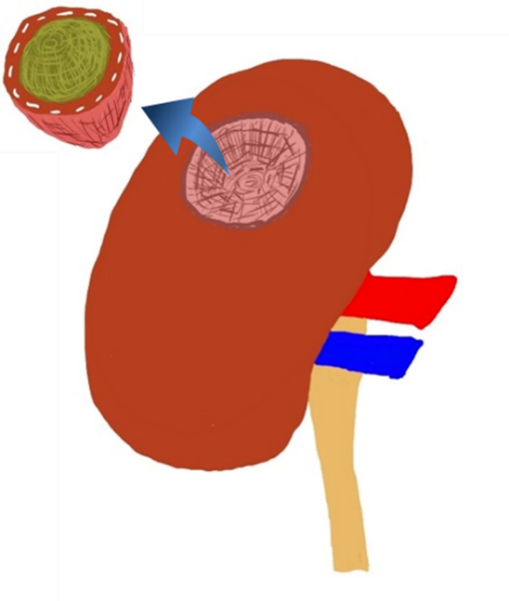


(c) 　　　　　　　　　　　　　　 (d)


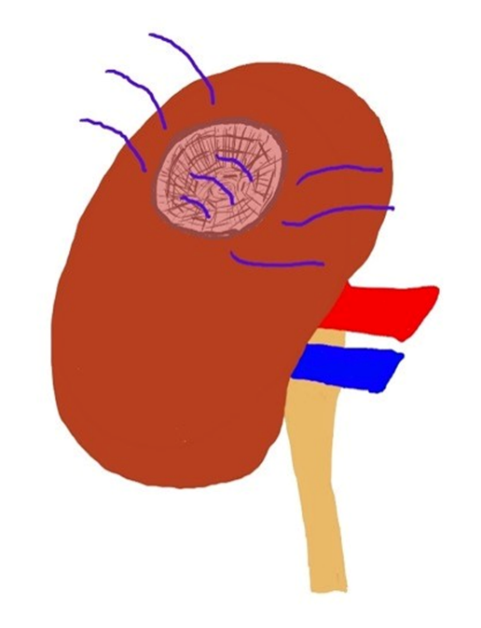
　　　　
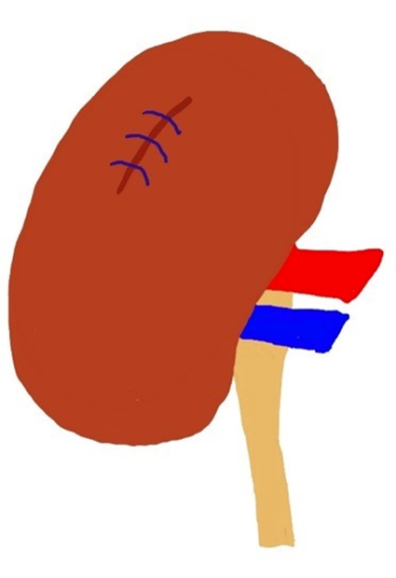

Supplement: S1 Fig — First, the blood flow to the affected kidney is temporarily blocked. Next, the tumor containing the surrounding normal tissue is excised, the cross-section of the extracted kidney is sutured hemostatically, and finally, blood flow is resumed. (a) Asterisk is renal tumors that protrude on the surface of the kidney. (b) The excised tumor containing the surrounding normal tissue. (c) Suturing of the cross-section of the extracted kidney. (d) Finished hemostasis of the defect. (DOCX) [file pone.0263179.s001.docx]
